# Clinical Ocular Exposure Extrapolation for a Complex Ophthalmic Suspension Using Physiologically Based Pharmacokinetic Modeling and Simulation

**DOI:** 10.3390/pharmaceutics16070914

**Published:** 2024-07-09

**Authors:** Maxime Le Merdy, Jessica Spires, Ming-Liang Tan, Liang Zhao, Viera Lukacova

**Affiliations:** 1 Simulations Plus, Inc., 42505 10th Street West, Lancaster, CA 93534, USA; 2 Division of Quantitative Methods and Modeling, Office of Research and Standards, Office of Generic Drugs, Center for Drug Evaluation and Research, U.S. Food and Drug Administration, 10903 New Hampshire Avenue, Silver Spring, MD 20993, USA

**Keywords:** PBPK, ocular PBPK, ophthalmic suspension, generic, besifloxacin

## Abstract

The development of generic ophthalmic drug products with complex formulations is challenging due to the complexity of the ocular system and a lack of sensitive testing to evaluate the interplay of its physiology with ophthalmic drugs. New methods are needed to facilitate the development of ophthalmic generic drug products. Ocular physiologically based pharmacokinetic (O-PBPK) models can provide insight into drug partitioning in eye tissues that are usually not accessible and/or are challenging to sample in humans. This study aims to demonstrate the utility of an ocular PBPK model to predict human exposure following the administration of ophthalmic suspension. Besifloxacin (Bes) suspension is presented as a case study. The O-PBPK model for Bes ophthalmic suspension (Besivance^®^ 0.6%) accounts for nasolacrimal drainage, suspended particle dissolution in the tears, ocular absorption, and distribution in the rabbit eye. A topical controlled release formulation was used to integrate the effect of Durasite^®^ on Bes ocular retention. The model was subsequently used to predict Bes exposure after its topical administration in humans. Drug-specific parameters were used as validated for rabbits. The physiological parameters were adjusted to match human ocular physiology. Simulated human ocular pharmacokinetic profiles were compared with the observed ocular tissue concentration data to assess the OCAT models’ ability to predict human ocular exposure. The O-PBPK model simulations adequately described the observed concentrations in the eye tissues following the topical administration of Bes suspension in rabbits. After adjustment of physiological parameters to represent the human eye, the extrapolation of clinical ocular exposure following a single ocular administration of Bes suspension was successful.

## 1. Introduction

Ophthalmic drug products are vital in treating various ocular diseases such as glaucoma, inflammation, and infection in human patients. The development of ophthalmic generic drug products encounters significant hurdles as the active pharmaceutical ingredients (APIs) reach the site of action before they enter the systemic circulation and usually produce very low to undetectable levels in the systemic circulation. Moreover, an in silico analysis demonstrated that the systemic pharmacokinetic (PK) metrics may not be sensitive to changes in formulations’ physicochemical properties [1]. Hence, in vivo bioequivalence (BE) studies with PK endpoints in plasma may not be appropriate or feasible for ophthalmic drug products [2]. Recognizing these challenges, the U.S. Food and Drug Administration (FDA) has ongoing research efforts to encourage generic drug development for locally acting drug products under the Generic Drug User Fee Amendments regulatory research program [3]. Through both internal and external research initiatives, the FDA seeks to either develop and validate new tools capable of supporting the development and regulatory assessments of ophthalmic generic drug products or enhance the scientific understanding of the interplay between a formulation and the ocular surface [4].

The complexity of the ocular system and the difficulty in accurately assessing its interaction with ophthalmic formulations pose significant challenges in the development of generic ophthalmic drug products. The FDA recommends a combination of in vitro characterization, in vivo pharmacokinetic (PK), pharmacodynamic (PD), and/or comparative clinical endpoint studies to demonstrate (BE) for these products. The determination of the appropriate study or combination of studies is based on the complexity of the dosage form (e.g., solution, suspension) and the scientific understanding of drug release and disposition in the eye [5]. However, conducting in vivo BE studies presents obstacles for the generic industry, including high costs, limited sensitivity to formulation differences in PD endpoints, substantial variability, and sparse sampling designs for PK studies, hampering the progress of generic ophthalmic drug development [6]. Per the Orange Book [7], most of the marketed ophthalmic generics are for ophthalmic solutions with a few others for ophthalmic suspensions. Therefore, gaps are limiting the development and regulatory acceptance of complex ophthalmic formulations. Addressing these challenges through further research is important to alleviate both developmental and regulatory hurdles in this field.

Initially introduced in the 1970s, physiologically based pharmacokinetic (PBPK) models have emerged as valuable tools supporting drug product development from preclinical to clinical trials, offering potential cost reductions and mitigating attrition rates [8,9]. Specifically, ocular PBPK (O-PBPK) models play a crucial role by offering insights into drug distribution within eye tissues that are either inaccessible or challenging to sample in human subjects, serving as an alternative approach to investigating the PKs and PDs of ophthalmic drug products. Leveraging the comparable physiology of rabbit eyes to that of humans [10], rabbit models are commonly utilized as the primary preclinical platform to assess the effects of changes in formulation attributes on APIs’ ocular exposure. This physiological similarity positions rabbits as the preferred species for the PBPK-based extrapolation of human ocular exposure based on preclinical data [10].

Starting in 2020, through grant 1U01FD006927, the Office of Generic Drugs at the FDA initiated a collaboration with Simulation Plus, Inc. to further advance O-PBPK by expanding the existing knowledge base for ocular drug absorption and disposition. This collaboration seeks to validate the ability of an O-PBPK model to predict ophthalmic clinical PK/PD based on PBPK models validated against preclinical data. To achieve this goal, an initial focus has been on studying ophthalmic solutions, with recent successful demonstrations of the O-PBPK model’s ability to predict their clinical PK from preclinical data [11].

This article describes the use of an O-PBPK model to predict local and systemic exposure, following the topical administration of a complex ophthalmic suspension once it was validated using ocular rabbit PK data. Besifloxacin (Bes) is a fluoroquinolone administered as an ophthalmic suspension to treat local infections on the surface of the eye. This API is used as a case study for this research project based on the availability of local ocular tissue concentrations in both rabbits and humans. This study includes (1) the development and validation of the ocular PBPK models for Bes in rabbits and (2) the prediction of clinical ocular exposure of Bes using the validated rabbit PBPK models.

## 2. Methods

### 2.1. Besifloxacin Drug Product

**Figure 1:**
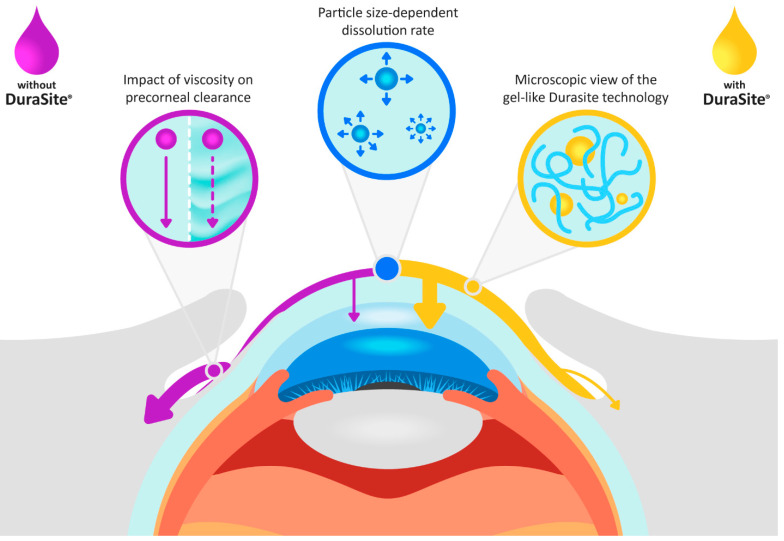
The effect of ophthalmic suspension CQAs and Durasite on besifloxacin flow on the surface of the eye and its absorption in the ocular tissues, compared to an ophthalmic suspension not formulated with Durasite. For the suspension without Durasite, an increase in viscosity reduces the elimination rate from the ocular surface (normal tear flow rate: solid purple line, reduced tear flow rate: dashed purple arrow).

Bes (drug product: Besivance^®^, Bausch + Lomb, Rochester, NY, USA) is a fluoroquinolone antimicrobial indicated for the treatment of bacterial conjunctivitis caused by susceptible isolates of numerous bacteria. Besivance composition is presented in Table 1. It is formulated as a topical ophthalmic suspension containing 6 mg of besifloxacin per milliliter (0.6%) [12]. The impact of ophthalmic suspension critical quality attributes (CQAs) (e.g., particle size, viscosity) on API ocular absorption has been extensively described in previous publications [1,13] (Figure 1). Additionally, this formulation utilizes Durasite^®^ (InSite Vision Inc., Alameda, CA, USA), a mucoadhesive vehicle engineered to extend the duration of a drug on the ocular surface. The key component of DuraSite is polycarbophil, a mucoadhesive polymer. Upon contact with tears, polycarbophil expands, forming substantial, stable, gel-like particles with heightened viscosity, making them less prone to being washed away by lacrimal drainage [14] (Figure 1).

### 2.2. Software and Model Structure

GastroPlus^®^ (version 9.9, Simulation Plus Inc., Lancaster, CA, USA) was used for the simulation of Bes biodistribution in rabbits and humans. The model structure combines a one-compartment model describing Bes systemic tissue distribution and clearance and the Ocular Compartmental Absorption and Transit (OCAT™) model (Version 3) describing Bes ocular drug absorption and disposition. In addition, to account for the intestinal absorption occurring to the fraction of the dose washed from the surface of the eye through nasolacrimal drainage, the Advanced Compartmental Absorption and Transit (ACAT^®^) model was used. The PBPK model parameters were obtained from multiple literature sources, estimated using built-in QSAR models in GastroPlus, or fitted based on in vivo rabbit data. All API-specific and formulation-specific parameters are presented in Table 2. A detailed description of the OCAT model structure and underlying equations describing ocular drug absorption and disposition have been presented in previous publications [1,13].

### 2.3. Parameterization of Besifloxacin Suspension Parameters

Two model settings were used to describe local and systemic Bes concentration following its topical ophthalmic administration.

Firstly, the dosage form was set as an ophthalmic suspension. The dissolution rate was calculated using the Lu, Frisela, and Johnson dissolution model [18]. As particle size in Besivance could not be identified in publicly available databases, a mean particle size diameter of three micrometers was assumed. This value is in line with other values reported for other ophthalmic suspensions [1]. This model setting does not account for the specific characteristics of Durasite on Bes residence time on the eye’s surface.

Therefore, in the second step, the observed data were modeled using a topical controlled release (CR) formulation. To perform this simulation, the water solubility of Bes salt was used to calculate the dissolved fraction in the Besivance drug product (16.7% dissolved). Then, a mixed multiple-dose support file was created so that 16.7% of the administered dose was given as an ophthalmic solution and 83.3% as an ophthalmic controlled release. Using this model setting, the Weibull function parameters describing Bes release from the CR formulation were simultaneously fitted based on ocular concentration–time courses.

For all simulations, the drainage rate constant was set to 0.1 min^−1^, to account for Besivance viscosity [19]. The pre-cornea maximum volume was set to 35 for rabbits and 37 µL for humans, reflecting the physiological tear volume (5 µL for rabbits and approximately 7 µL in humans) plus the standard 30 µL administered volume of a topical ophthalmic drug product. Consequently, if more than 30 µL is administered in a preclinical or clinical study, the OCAT model automatically eliminates the excess fluid. During simulation, after the administration of the suspension eye drops, the pre-cornea compartment volume gradually decreases to the physiological tear volume via nasolacrimal drainage. For simulations using the suspension dosage form, this process removes both dissolved and undissolved drugs from the pre-cornea compartment, preventing their absorption in the cornea or conjunctiva. The OCAT model considers evaporation to be negligible.

### 2.4. Ophthalmic PK Clinical Extrapolation Strategy

Table 3 presents the preclinical and clinical studies used to develop and validate the OCAT model in rabbits and perform the clinical PK extrapolation. Preclinical PK data in albino New Zealand white (NZ) rabbits from two studies were available. One study was used to develop the baseline OCAT model (study Bes.NZ.1). The initial simulation was performed with all default OCAT parameters and the available NZ ocular physiology in GastroPlus version 9.9. The predictions were compared with observed data to assess model performance. To address the mispredictions, a minimal number of OCAT parameters were fitted. The model was considered acceptable if the simulated ocular tissue concentration–time courses could be overlaid with observed data. The adjusted parameters were validated by predicting ocular PK for additional studies in NZ (Bes.NZ.2) and Dutch Belted (DB) rabbits (studies Bes.DB.1 and Bes.DB.2) using corresponding physiologies available in GastroPlus version 9.9 (external model validation).

The validated model was subsequently applied to predict ocular PK in humans. API and CR formulation-specific parameters retained the same values as those fitted to rabbit ocular PK data. The PBPK model’s physiological parameters were adjusted to align with human ocular physiology by utilizing the default human ocular physiology settings in GastroPlus. The dose, dose volume, and dose administration schedule were set according to clinical study protocols. The simulated human ocular PK profiles were then compared with clinically observed concentration data to evaluate the OCAT model’s accuracy in predicting human ocular exposure through interspecies extrapolation.

## 3. Results

### 3.1. Rabbit OCAT Models

Two preclinical studies in NZ rabbits and two in DB rabbits with reported Bes ocular tissue concentrations were identified in the literature (studies’ codes: Bes.NZ.1 to Bes.DB.4; data sources and study protocols are listed in Table 3). Bes.NZ.1, which lists tears, cornea, conjunctiva, aqueous humor (AH), and plasma concentration–time courses, was used for model development.

**Figure 2:**
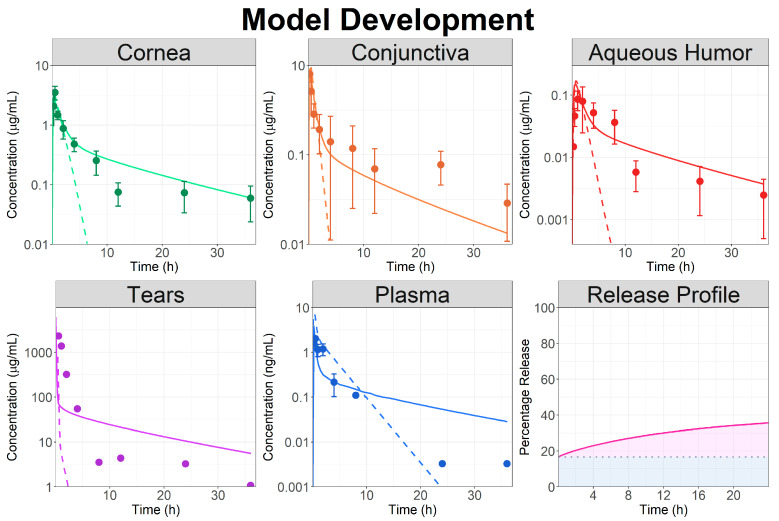
Model development: observed (circles) and simulated (lines) ocular time courses in NZ rabbits following a single topical administration of besifloxacin suspension at 0.6% (study Bes.NZ.1). Dashed lines represent model simulation using the topical ophthalmic suspension dosage form and solid lines represent the model simulation using the topical ophthalmic controlled released dosage form. The fitted Weibull function describing the besifloxacin release profile from the controlled release dosage form is also presented. The blue shaded area represents the percentage of the dose administered as an ophthalmic solution whereas the pink shaded area represents the fraction released from the controlled release formulation.

Initial simulation was performed using a drainage rate of 0.1 min^−1^ to account for Besivance viscosity and a particle size diameter of 3 µm. The cornea epithelium and conjunctiva permeabilities were fitted to 1 × 10^−7^ and 1.4 × 10^−7^ cm/s, respectively. For all the other ocular tissues, the permeabilities were kept at default values (estimated by GastroPlus from the Bes physicochemical properties). The ICB systemic absorption rate constant was also fitted (5 × 10^−3^ 1/s). The model reasonably captured the observed data for NZ rabbits, but only for the first hours, and underpredicted observed concentrations after 5 h (Figure 2, dashed lines). Additional model parameter adjustments were attempted to account for the effect of Durasite on local Bes exposure. Yet, all those attempts failed to accurately describe the entirety of the observed ocular concentration–time courses.

Therefore, the dosage form in the model was changed to a combination of the topical ophthalmic solution and controlled released formulation. To describe both Bes dissolution in the tears and its release from the gel form by Durasite, a single Weibull function was used. The Weibull parameters (Table 2) were fitted based on observed ocular tissue data and model results are presented in Figure 2 (solid lines). Using a CR formulation allowed an accurate description of Bes local and systemic PK. The model simulation indicated that only 20% of the dose administered as CR (83.3% of the total administered dose) was released in the first 24 h to describe the observed data.

**Figure 3:**
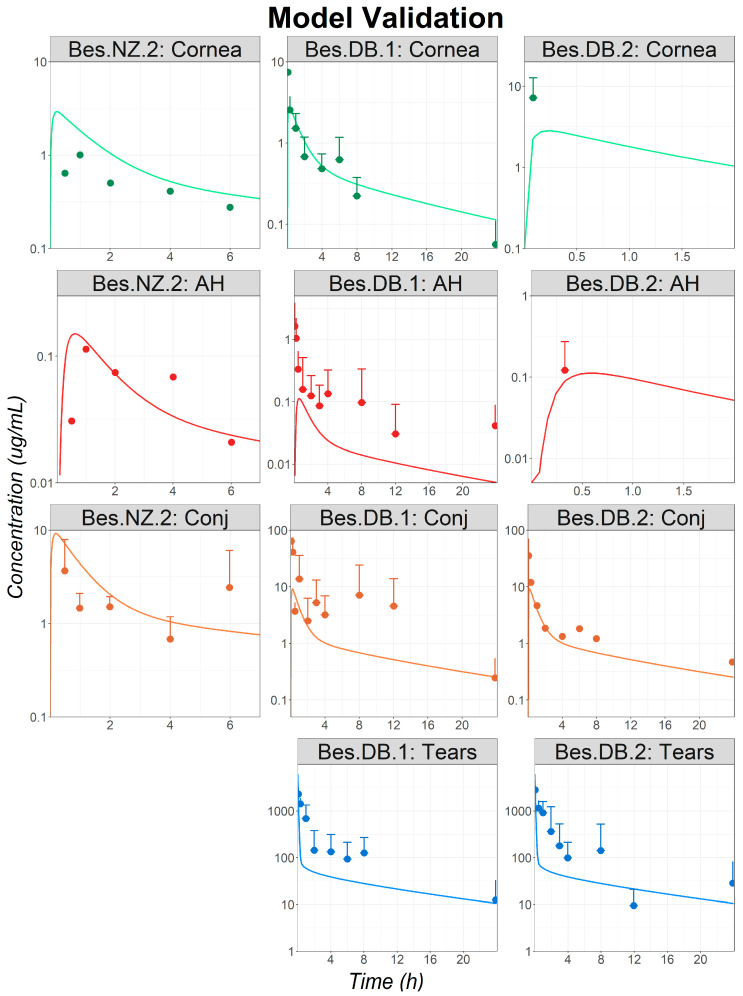
Model validation: observed (circles) and simulated (lines) ocular time courses in NZ and DB rabbits following a single administration of besifloxacin suspension at 0.6% (studies Bes.NZ.2, Bes.DB.1, and Bes.DB.2). Only the means + standard deviations (SDs) are represented for all studies for which the SDs were reported as, in most cases, the SDs are superior to the reported mean values and the means − SD could not be represented in the logarithmic scale. Simulations were performed using the controlled release dosage form. Abbreviations—AH: Aqueous Humor; Conj: Conjunctiva.

The fitted parameters (Bes ocular permeabilities, and release profile) were validated by predicting the ocular concentration–time courses following the administration of a Bes suspension to both NZ and DB rabbits (studies: Bes.NZ.2, Bes.DB.1, and Bes.DB.2). Final simulations for ocular concentration–time courses are presented in Figure 3. Ocular concentrations following the single-dose administrations of Bes suspensions were well predicted for all studies. Hence, the external validation was completed, and the model was deemed acceptable for a further analysis and to predict Bes human ocular exposure.

### 3.2. Ophthalmic PK Clinical Extrapolation

Clinical extrapolation was conducted using the validated OCAT model. Both Bes and formulation-specific parameters from rabbit simulations were used. The physiological parameters were adjusted to reflect human ocular physiology. In addition, the dose, dose volume, and dosing regimen were set according to the published protocols for each human trial. Clinical data were collected from healthy subjects or patients undergoing cataract surgeries. Based on previous work, it was demonstrated that cataract surgery does not impact the ocular PK of fluoroquinolone topical solutions [11]. Hence, the same human ocular physiology was used for all studies. To evaluate the OCAT model’s accuracy in predicting human ocular exposure, individual simulations were performed for each study. Simulation results were compared with the corresponding observed data.

**Figure 4:**
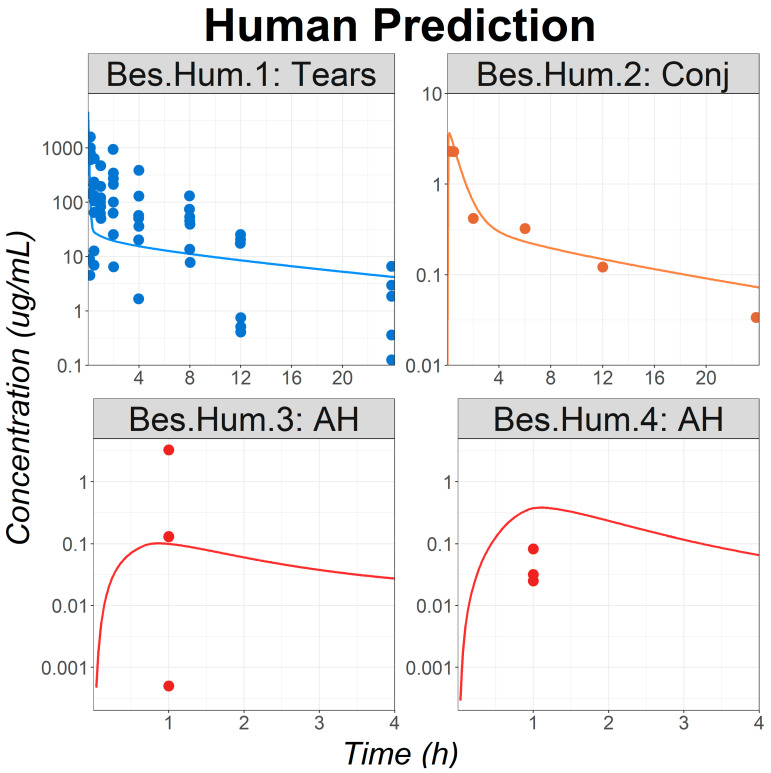
Model application: observed (circles) and simulated (lines) ocular time courses in humans following a single or multiple administrations of besifloxacin suspension at 0.6% (studies Bes.Hum.1 to Bes.Hum.4). Abbreviations—AH: Aqueous Humor; Conj: Conjunctiva.

For all studies describing Bes PKs following a single topical administration, the simulated profiles adequately described the observed data, especially in the tears and conjunctiva, both being the Bes site of action (Figure 4). Observed data were significantly overpredicted for the study Bes.Hum.4. It is the only study presenting data following multiple administrations of Bes. Therefore, it cannot be concluded yet if the cause of the misprediction is due to the study protocol, or to the known significant interstudy variability for clinical trials measuring local concentrations [11]. Nevertheless, it should be pointed out that the observed and simulated aqueous humor concentrations for study Bes.Hum.4 are within the observed concentration range of study Bes.Hum.3.

## 4. Discussion

An understanding of the ocular absorption mechanism is necessary for both the pharmaceutical industry and regulatory agencies to support the development and evaluation of new and generic drug products. Previous research projects described the methodology to develop and validate an OCAT model for rabbit physiology [1] and how those models can be used to predict clinical local exposure for ophthalmic solutions [11]. The OCAT model’s ability to predict ocular exposure in humans, once validated against rabbit data, has only been demonstrated for ophthalmic solution drug products [11]. The current study builds upon the work on solutions and demonstrates the ability of the OCAT model to predict human ocular exposure for a topical suspension. This is achieved by performing PK extrapolation using a validated PBPK model based on rabbit data for Bes.

The main difference between an ophthalmic topical solution and a suspension is the presence of suspended particles of API in the formulation. Those particles can have different shapes, sizes, and size distributions based on the suspension’s manufacturing process. In vivo studies demonstrated how particle size impacts the ocular absorption of dexamethasone [27], fluorometholone [28,29], and indomethacin [30], following their topical administration in rabbits. These data were used to integrate and validate the Bes particle size as a CQA in the OCAT model [1,31]. Furthermore, ophthalmic suspension’s viscosity was described as one of the key players in the complex interplay between API retention and dissolution in the pre-corneal compartment, thereby impacting ocular drug absorption [30]. In the model, an increase in the formulation’s viscosity reduces the tear drainage rate [32] and causes a decrease in the elimination rate of the suspension in the pre-corneal compartment. The OCAT ability to predict the effect of viscosity on local concentration has already been demonstrated for dexamethasone in rabbits [1]. This validated O-PBPK model integrating both the impact of particle size and viscosity was first used to predict Bes local and systemic exposure in rabbits. Even after optimizing some of the permeability parameters, the model could not capture the observed data. A realistic particle diameter was hypothesized as Bes particle size distribution could not be identified in the public domain. To test this hypothesis, a parameter sensitivity analysis testing the diameter was performed but was unsuccessful in capturing the observed data, especially in the elimination phase.

Hence, it was concluded that the miss prediction could only come from one of the Besivance characteristics, being the presence of Durasite in the formulation. Durasite is based on polycarbophil, an amphipathic polymer of polyacrylic acid. This excipient has been extensively described for its bioadhesive capacities and its usefulness as a support matrix for sustained drug release. Indeed, following topical administration, polycarbophil expands, forming a gel-like layer with heightened viscosity on the ocular globe surface. Additionally, in vitro and in vivo studies have demonstrated that polycarbophil binds certain neutral, cationic, and anionic small molecules, such as Bes, and then releases them over some time in a predictable manner [33]. Yet, the interaction between dissolved and undissolved Bes, the polymer layer, and tear drainage remains uncertain. Hence, to describe Bes local and systemic exposure following the topical administration of Bes, a topical CR formulation set-up was used in the developed OCAT model. The fitted release profile lumps Bes dissolution, release from the polymer layer, and the tear-mediated elimination (represented by the unreleased fraction). To improve the model and mechanistically describe Bes local tissue PK, having access to Besivance CQAs will be a critical factor.

For reliable model predictions, the Bes fraction dissolved in the formulation before the administration was shown to be of importance. In the simulations, Bes hydrochloride water solubility (1 mg/mL) was used to estimate the fraction dissolved in the drug product as it is present under the Bes salt form in the drug product [12]. This hypothesis may be challenged as a report calculated that only 1.5% of the drug substance is in the solution in the drug product [17], indicating a solubility of 0.09 mg/mL. However, when this solubility is implemented in the OCAT model, observed tears’ maximum concentration was largely underpredicted for all rabbit and human studies, using the suspension dosage form. To describe the observed tears’ data using the suspension dosage form, the solubility within the drug product was adjusted to 2.5 mg/mL, indicating that 41.66% of the administered dose would be dissolved in the drug product. This value is significantly higher than the one reported for Bes salt [12] or the one indicated in a report [17]. Therefore, due to the uncertainty of this model parameter, the observed in vitro value for the salt form [16] was fixed and the release profile was subsequently fitted using the controlled release dosage form. It is expected that if a different Bes solubility value were to be used, the ratio of dissolved and undissolved Bes would be affected, resulting in a different fitted release profile. Thus, until Bes solubility in the dosage form is determined (experimentally), the fitted release profile presented in the result section may not be a true representation of the in vivo release. Nevertheless, as the same model settings were used for all preclinical and clinical studies, the ability of the model to predict Bes exposure across multiple rabbit strains and different species was validated.

In both preclinical and clinical studies, a significant interstudy variability is observed. Potential causes (e.g., anesthetic agents) of the variability have previously been discussed [11]. However, for the Besivance case study, one clinical study (Bes.Hum.4) was largely overpredicted by the model, even though the observed aqueous humor concentrations for this study are comparable to observed data for study Bes.Hum.3. The Bes.Hum.4 study is the only one in which patients received multiple administrations of Besivance within a short period (one administration every ten minutes for a total of four administrations) [26]. Ten minutes is typically sufficient to wash out most of the administered volume from the surface of the eye. Yet, due to the combined effect of Besivance’s high viscosity and Durasite, it is plausible that after 10 min, most of the administered dose remains on the ocular surface, covering the entirety of the cornea and limiting the effect of subsequent administration. To test the hypothesis, a simulation was conducted for a single Bes administration and compared with observed data. The model still overpredicted the data but the prediction was within 3-fold, aligned with the typical observed intra- and interstudy variabilities.

Bes belongs to the fluoroquinolone drug family. In a previous research project [11], the ocular PBPK model was developed for three other fluoroquinolones administered as ophthalmic solutions: levofloxacin, moxifloxacin, and gatifloxacin. In all cases, melanin binding had to be implemented in the model to accurately describe local concentration in both albinos and pigmented rabbits. For those fluoroquinolones, the fitted melanin fraction unbound was fitted based on observed data in pigmented rabbits, and values between 1 and 0.1% were used in the final models [11]. For Bes, no observed data could be identified in the public domain reflecting its PK in ocular tissues containing melanin. Therefore, melanin binding could not be reliably parameterized in the Bes ocular PBK model. Furthermore, using one of the values previously determined for one of the other fluoroquinolones could increase model uncertainty as fluoroquinolones’ physicochemical parameters can largely vary between APIs [34].

In conclusion, the development and validation of an ocular PBPK model for an API administered as an ophthalmic suspension were performed based on preclinical data. This model was successfully applied to predict clinical exposure in multiple ocular tissues. This proposed methodology to extrapolate human ocular PK based on a validated PBPK model using preclinical data may support drug development and provide a better understanding of the impact of formulation characteristics on the in vivo performance of ophthalmic suspension products. A deeper understanding of key physiological mechanisms influencing PK outcomes as well as further extrapolation from rabbit to human models for other ophthalmic dosage forms are the next steps planned for this OCAT model.

## Figures and Tables

**Table 1:** Besivance^®^ composition. The list of chemicals was extracted from the drug product’s FDA label [12].

Chemical	Role
Besifloxacin	Active pharmaceutical ingredient
Polycarbophil	Bioadhesive support matrix
Mannitol	Diluent, Tonicity agent
Poloxamer 407	Wetting agent, Viscosity enhancer
Sodium chloride	Tonicity agent
Edetate disodium dihydrate	Preservatives, Chelating agent
Sodium hydroxide	Buffering agent
Water	Volume for injection
Benzalkonium chloride	Preservative

**Table 2:** Model Parameter Values Implemented in the O-PBPK Model for *Besifloxacin*.

Parameter	Definition	Units	Besifloxacin	
*Physicochemical Properties*			*Value*	*Source*
MWt	Molecular weight	g/mol	393.85	AP10.4 *
logP(neutral)	Log octanol/water partition coefficient	-	0.26	AP10.4
F_u_	Plasma unbound percent	%	41.5	[15]
F_u melanin_	Percent unbound to melanin	%	100	GP **
R_bp_	Blood-to-plasma-concentration ratio		1.33	AP10.4
Solubility	Maximum amount dissolved in water ***	µg/mL	1 (pH 7)	[16]
pKa	Acidity constant	-	1.8/6/9.9	[17]
			Base/Acid/Base	
Peff	Intestinal permeability	×10^−4^ cm/s	0.39	AP10.4
*Systemic parameters*				
Vc	Rabbit volume of distribution	L/kg	1.62	AP10.4
CL	Rabbit systemic clearance	L/h	15.42	AP10.4
OCAT^™^ *parameters*				
Perm_Cornea_epi_	Cornea epithelium permeability	×10^−7^ cm/s	1	fitted
Perm_Cornea_str_	Cornea stroma permeability	×10^−5^ cm/s	1.86	GP
Perm_Conjunctiva_	Conjunctiva permeability	×10^−7^ cm/s	1.4	fitted
Perm_AH_.	Aqueous humor permeability	×10^−6^ cm/s	8.51	GP
Perm_ICB_	Iris–ciliary body permeability	×10^−4^ cm/s	7.74	GP
Perm_Sclera_	Sclera permeability	×10^−5^ cm/s	1.02	GP
Perm_Choroid_	Choroid permeability	×10^−4^ cm/s	1.84	GP
Perm_Retina_	Retina permeability	×10^−5^ cm/s	1.73	GP
Perm_V_._H_.	Vitreous humor permeability	×10^−6^ cm/s	6.7	GP
SAR_Choroid_	Choroid systemic absorption rate	×10^−4^ s^−1^	2.75	GP
SAR_Retina_	Retina systemic absorption rate	×10^−3^ s^−1^	1.2	GP
SAR_Conjunctiva_	Conjunctiva systemic absorption rate	×10^−4^ s^−1^	3.81	GP
SAR_ICB_	Iris–ciliary body systemic absorption rate	×10^−3^ s^−1^	5	fitted
*Formulation parameters*				
PS	Suspended particles’ mean diameter	µm	3	assumed
Max	Weibull total released parameter	%	25.47	fitted
A	Weibull time scale	h^b^	11.93	fitted
b	Weibull shape	-	0.88	fitted

*: AP10.4 refers to ADMET Predictor^®^ version 10.4. **: GP refers to GastroPlus default parameters. ***: Value reported for the salt besifloxacin hydrochloride.

**Table 3:** List of preclinical and clinical studies with available ocular tissue concentrations used for Besifloxacin OCAT model development and validation.

Study Code	Species	Conc (%W/V)	Dose	Volume (µL)	Tissue of Interest	Source
Preclinical Studies						
Bes.NZ.1	NZ	0.6	single	50	Cornea, Conj, AH, Tears, Plasma	[20]
Bes.NZ.2	NZ	0.6	single	50	Cornea, Conj, AH	[21]
Bes.DB.1	DB	0.6	single	50	Cornea, Conj, AH, Tears	[22]
Bes.DB.2	DB	0.6	single	50	Cornea, Conj, AH, Tears	[23]
Clinical Studies						
Bes.Hum.1	HS	0.6	single	50	Conj	[24]
Bes.Hum.2	HS	0.6	single	50	Tears, Plasma	[22]
Bes.Hum.3	CP	0.6	single	50	AH	[25]
Bes.Hum.4	CP	0.6	multiple	50	AH	[26]

NZ = New Zealand Rabbit; DB = Dutch Belted Rabbit; HS = Healthy Subjects; CP = Patients with Cataract; Conj = Conjunctiva; AH = Aqueous Humor; Conc = Concentration.

## Data Availability

All data are published and listed in the paper.
